# Evaluating the Impacts of Methylsulfonylmethane on Allergic Rhinitis After a Standard Allergen Challenge: Randomized Double-Blind Exploratory Study

**DOI:** 10.2196/11139

**Published:** 2018-11-29

**Authors:** Susan Hewlings, Douglas S Kalman

**Affiliations:** 1 Department of Nutrition Central Michigan University Mount Pleasant, MI United States; 2 Nova Southeastern University Health Professionals Division, Exercise Science Davie, FL United States

**Keywords:** methylsulfonylmethane, sulfur, allergies, allergic rhinitis, supplements

## Abstract

**Background:**

The sulfur-containing compound methylsulfonylmethane (MSM) has been used as a dietary supplement for a variety of reported health benefits. Clinical observations and case studies have indicated that MSM may help alleviate allergic rhinitis; however, this effect has not been evaluated under controlled conditions.

**Objective:**

This study aimed to determine the effects of MSM consumption on allergic rhinitis symptoms after provocation with a standardized allergen.

**Methods:**

We recruited healthy participants with a history of allergic nasal congestion to participate in a randomized, double-blind, adaptive-design study. Participants were administered a standardized allergen in clinic to determine the presence or absence of an allergic response. Participant responses were recorded using a recognized measure of nasal patency, peak nasal inspiratory flow (PNIF), and by a visual analog scale to score the severity of their allergy-related nasal symptoms. After we collected baseline nasal responses to allergen, followed by a 1-week washout period, participants returned to the clinic and were exposed to allergen after taking an acute high dose of 12 g of MSM. We then randomly assigned participants to a lower dose of MSM (1 g, 3 g, or 6 g), which they consumed once a day for 14 days. Participants returned to the clinic for repeat assessments while again taking their assigned daily dose of MSM.

**Results:**

All MSM treatment courses significantly reduced visual analog scale average nasal symptoms in a longitudinal comparison across all participants, with low-dose treatments decreasing symptoms by 53.72% (*P*=.001), and an acute 12-g dose decreasing symptoms by 22.49% (*P*=.03). Although the acute dose of MSM did not yield significant changes in nasal patency, low “everyday” doses significantly relieved nasal obstruction as indicated by a 17.32% (*P*=.02) increase in PNIF across all participants. The most effective dose across all measurements was daily consumption of 3 g of MSM, which significantly decreased all nasal symptoms (nasal obstruction, rhinorrhea, watery or itchy eyes and nose, and sneezing) and further was found to significantly (*P*=.01) increase PNIF.

**Conclusions:**

The MSM study product provided significant relief of allergic rhinitis symptoms and objective nasal obstruction measurements without the occurrence of adverse events. Oral consumption of the study product may reduce the symptoms and onset of allergic rhinitis without the side effects associated with standard-care medication.

**Trial Registration:**

ClinicalTrials.gov NCT02342483; https://clinicaltrials.gov/ct2/show/NCT02342483 (Archived by WebCite at http://www.webcitation.org/73vLKNvAp)

## Introduction

### Background

Allergic rhinitis, or nasal allergy, is an extremely common ailment that occurs when the nasal mucosa undergoes inflammation in response to inhaled allergens [1]. The prevalence of this condition ranges from 10% to 20% in the United States [2]. It is characterized by several uncomfortable symptoms: red itchy eyes; a blocked, itching, runny nose (a group of symptoms referred to as rhinorrhea); and sneezing. Other reported symptoms include throat clearing, headaches, facial pain, ear pain, itchy throat and palate, snoring, and sleep disturbances. Severe allergic rhinitis can significantly affect the patient’s quality of life, sleep, and work performance [3]. Nasal allergy commonly occurs when an individual’s immune system overreacts to allergens such as grass, weed, or tree pollens; house dust; mites; mold; and animal dander [4,5].

Allergic rhinitis was previously considered to be a disorder localized in the nasal passages, but emerging research indicates that the entire respiratory tract is involved. Close physiological, functional, and immunological relationships exist between the upper (nose, nasal cavity, paranasal sinuses, pharynx, and larynx) and lower (trachea, bronchial tubes, bronchioles, and lungs) respiratory tracts [6]. Although allergic rhinitis can be considered a simple nuisance in the case of mild symptoms, it has been classified as a chronic disease that should be addressed by a physician [7]. Allergic rhinitis is also associated with serious inflammatory disorders, including asthma. In fact, 80% of asthmatic patients have allergic rhinitis and 40% of rhinitis patients have asthma [8,9]. Allergic rhinitis is controlled by various palliative therapies, most commonly antihistamine medications, which often produce sedative side effects [10].

Methylsulfonylmethane (MSM), also known as dimethyl sulfone and methyl sulfone, is an organic compound containing sulfur that occurs naturally in the body, as well as in a variety of fruits, vegetables, grains, and animals [5]. Orally and topically, it is used to treat chronic and musculoskeletal pain, osteoarthritis, joint inflammation, exercise-induced muscle damage, hemorrhoids, and rosacea [11]. However, only a few studies have reported the potential benefits of MSM in treating allergic rhinitis and allergic sinusitis [12,13].

One multicenter human trial found that the consumption of 2.6 g of MSM effectively reduced symptoms of seasonal allergic rhinitis (SAR). This dose of MSM improved the frequency of upper respiratory signs and symptoms such as runny nose, nasal obstruction, and paroxysmal sneezing after a week of oral intake [13]. However, that study prompted criticism because it lacked quantification of pollen count each participant was exposed to [14]. In this study, we aimed to address the efficacy of MSM treatment using controlled standardized conditions in healthy participants with a history of SAR.

MSM is considered safe for consumption, as clinical studies have reported few, if any, side effects in a human population [13]. Moreover, in rats, MSM administered at 2 g/kg, a dose 5 to 7 times the maximum recommended dose for humans, was well tolerated and elicited no adverse events or deaths. No gross pathological lesions or changes in organ weights were observed, and renal history appeared to be normal in treated rats [12]. Similarly, oral intake of MSM also led to no adverse events in pregnant rats [15], suggesting it is safe for consumption even at high doses.

### Objective

This randomized, double-blind, adaptive-design study aimed to assess the efficacy of the MSM study product in attenuating nasal provocation after exposure to standardized allergens. End points were percentage change in peak nasal inspiratory flow (PNIF) and visual analog scale (VAS) nasal symptom score in response to allergen exposure.

## Methods

### Investigational Product

The investigational product for this study was OptiMSM (Bergstrom Nutrition, Vancouver, WA, USA, the sponsor of this study). OptiMSM is designated “generally recognized as safe” with a letter of no questions issued by US Food and Drug Administration Center for Food Safety and Applied Nutrition (USFDA-CFSAN). Doses for study evaluation were chosen by the sponsor (1 g, 3 g, 6 g, and 12 g). The study products were provided by the sponsor and were consumed once a day, according to the randomization assignment.

### Participants

We recruited study participants through online or database recruitment and screened them by telephone prior to scheduling a screening visit. We enrolled and randomly assigned healthy volunteers into the study who were between the ages of 18 and 65 years who had a history of nasal congestion in response to pollen, dust mites, cat dander, or dog dander and who scored moderate or severe in the visual analog scale (VAS) for nasal symptoms in response to an allergenic challenge at screening (V1). Pregnant or lactating women and participants with idiopathic rhinitis, atrophic rhinitis, or rhinitis medicamentosa were excluded from the study. Those taking any antihistamine or antiallergenic products underwent a 1-week washout period of these medications.

### Study Design

This study was a randomized, double-blind, adaptive-design clinical trial with a duration of up to 14 days. Group allocation was placed in individually numbered envelopes to maintain blinding of all individuals. The participants, as well as the clinical staff, data management staff, and statistical analysis staff, were unaware of the study group. This study was conducted by a contract research organization (Medicus Research, Northridge, CA, USA). The study and the informed consent were approved and monitored by the MaGil Institutional Review Board (Rockville, MD, USA) prior to the initiation of any study-related activities.

The study duration was 14 days, with a total of 3 visits: V1 (screening visit), V2 (administration of the acute dose of MSM of 12 g), and V3 (administration of varying doses of MSM of 1 g, 3 g, or 6 g; end of the study). After V2, participants were randomly assigned to receive 1 of 3 doses of MSM (1 g, 3 g, or 6 g) to consume daily for 14 days; they then returned to the clinic at V3 for repeat assessments in response to the allergen challenge.

For the initial screening visit, participants were acclimated in the room for 1 hour to allow for a washout of environmental allergens. All participants underwent the informed consent process and were screened for the presence of all the inclusion criteria and the absence of all the exclusion criteria. The screening process also included a detailed medical history, prior and concomitant medications, a physical examination, and measurement of vital signs. Participants also had laboratory assessments, including a urine pregnancy test. To determine the presence or absence of response, participants were administered an allergenic challenge consisting of the aerosolized allergens listed in Textbox 1 (supplier: Jubilant HollisterStier, LLC, Spokane, WA, USA).

In the study, the dose of allergen was set at 10,000 bioequivalent allergy units/mL, an allergen dose that has been shown to result in a substantial drop in average PNIF based on a study of Scadding et al [16].

After exposure to an allergen, participants had their PNIF measured and answered the VAS nasal symptom score questionnaire at multiple time points over 1 hour: –30 minutes (preexposure), 5 minutes (postexposure), 15 minutes (postexposure), 30 minutes (postexposure), and 60 minutes (postexposure). The allergen was administered at T0. Participants were provided with a rescue dose of medication (50 mg diphenhydramine [Benadryl]) if they continued to experience severe symptoms at T60 minutes.

After a 1-week washout period, participants returned to the clinic for V2, when they were interviewed by clinical staff to determine changes in medical history or the start of any new medications. After adverse event review and retaking of vital signs, participants were randomly assigned to 1 of the 3 test doses. Participants consumed an acute dose of the study product (MSM 12 g) 30 minutes prior to allergen exposure. At T0, the allergen was administered. Following exposure, participants’ PNIF and VAS nasal symptom score were recorded at multiple time points over the following 2 hours: –30 minutes (preexposure), 15 minutes (postexposure), 30 minutes (postexposure), 60 minutes (postexposure), and 120 minutes (postexposure). At the end of V2, participants were dispensed with a 14-day supply of study product along with a daily dosing diary and a rescue medication (diphenhydramine 50 mg) to be used if they experienced severe symptoms.

Participants returned to the clinic for V3, when they were interviewed by clinical staff to determine changes in medical history and screened for adverse events. Participants were assessed for compliance by review of completed paper diaries and assessment of leftover and used study products. Participants were again exposed to allergen and received their randomized study product dose (MSM 1 g, 3 g, or 6 g). PNIF and VAS nasal symptom scores were recorded on a similar schedule to that at V2**)**. Rescue medication was offered if they continued to experience severe symptoms.

**Allergen exposure protocol: aerosolized allergens.**

Pollen

- Tree pollen
  - Oak mix
  - Birch mix
  - Mountain cedar
  - Pecan
- Grass pollen
  - Bermuda grass
  - Kentucky bluegrass
  - Fescue, meadow
  - Johnson grass
  - Ryegrass, perennial

Dust mites

Cat dander

Dog dander

### End Points

The objectives of this study were to assess the efficacy of MSM in improving nasal breathing and promoting recovery of nasal breathing, and improving “stuffy nose” symptoms after exposure to environmental allergens. The end points for this objective were percentage change from allergen exposure to each time point in PNIF and VAS nasal symptom scores (including nasal obstruction, rhinorrhea, watery eyes, itching eyes, itching nose, and sneezing).

### Statistics

Parallel dual data entries were done by data management personnel across all end points. Data were validated and parallel entries were reconciled after the dual data entry process. The monitoring team compared the values on the original source documents, correcting any discrepancies found. All data elements were screened for reasonableness, and all missing, suspicious, or impossible values were referred back to the monitoring team for query generation and resolution. The database was formally locked after all suspicious entries in the database were resolved. The product assignments were then distinguished from the randomization or blinding codes and merged into the database and data tables.

We processed descriptive measures such as numbers, means, standard deviations, and standard errors of means for each numeric end point on all visits. Percentage changes were used to quantify increase or decrease of end points from baseline for each arm. On the other hand, categorical end points were presented as frequency tables, with corresponding percentages.

We performed a modified per-protocol analysis to assess the efficacy variables of the study. Participants who completed at least one postdose visit were included in the analysis. All efficacy end points were analyzed depending on the level of measurement of the end point.

For each end point in the interval or ratio scale that followed a normal distribution (or had semblance to normality), we analyzed the data using a paired *t* test for comparison between visit 1 and other time points. For nonnormally distributed data, we performed a sign test to analyze changes from each MSM product. Lastly, in longitudinal data, we measured the dependent variable at several time points for each participant and analyzed using a linear mixed model. In the analysis, the different doses of the MSM product were the factor, and the value of the efficacy variable at every visit was modeled as a function of group (response variable of interest) and of the value of the efficacy variable readings in every time point (covariate).

The linear mixed model procedure expands the general linear model so that the error terms and random effects are permitted to exhibit correlated and nonconstant variability. The linear mixed model, therefore, provides the flexibility to model not only the mean of a response variable, but its covariate structure as well. We selected categorical predictors as factors in the model. Each level of a factor can have a different linear effect on the value of the dependent variable. We selected scale predictors as covariates in the model. Within combinations of factors levels, values of covariates are assumed to be linearly correlated with values of the dependent variable. Repeated-effects variables are variables whose values in the dataset can be considered as markers of multiple observations of a single participant. Participant variables define the individual participants of the repeated measurement. In addition, the model used in the 1-way analysis of variance procedure was equivalent to fitting a linear mixed model with 1 fixed factor. All tests of hypotheses were done at alpha=.05.

To obtain comparable documentation on adverse events, the investigator asked each participant open, standardized questions at each visit. The frequency and intensity of adverse events and serious adverse events were recorded in detail, based on the participant’s interviews during each visit. We grouped recorded adverse events by general type of event (body system). We assessed differences in adverse event patterns for each MSM product dose by McNemar change test.

## Results

### Participant Allocation

**Figure 1:**
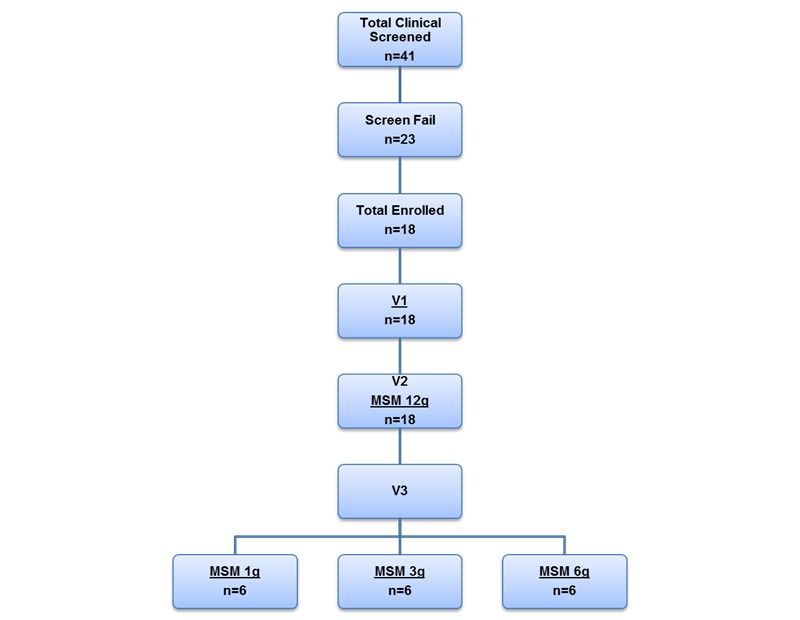
Attrition chart for the study. MSM: methylsulfonylmethane; V1: visit 1, screening visit; V2: visit 2; V3: visit 3.

Of the 41 participants screened for this study, 18 passed the screen, and all 18 were retained through completion of the clinical trial. The 18 participants attended an initial screening visit (V1), when baseline responses to allergen exposure were recorded. At V2, all 18 participants were exposed to allergen after consumption of MSM 12 g, randomly assigned to 3 groups of 6 participants each, and then given a 14-day supply of their assigned randomized study product (MSM 1 g, 3 g, or 6 g) to self-administer once daily. At V3, participants were again assigned to consume differing amounts of MSM before and after exposure to allergen (MSM 1 g, 3 g, or 6 g; Figure 1).

### Longitudinal Comparisons

**Figure 2:**
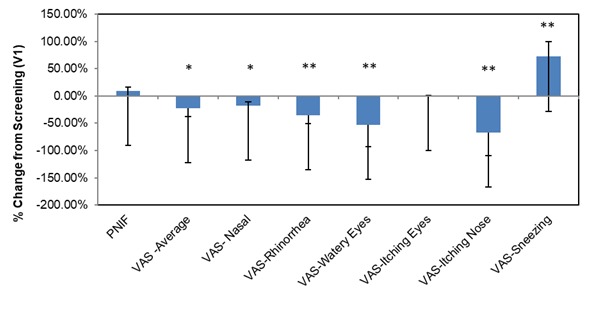
Methylsulfonylmethane (MSM) 12g percent change from screening. Peak nasal inspiratory flow (PNIF) and visual analog scale (VAS) nasal scores show the effects of an acute high methylsulfonylmethane dose (12 g) on patient symptoms. Significance was tested using linear mixed model analysis. *P ≤.05; **P ≤.01.

An acute dose of 12 g of MSM significantly decreased the VAS average nasal symptom score by 5.95 U from screening to baseline (22.49%; *P*=.03) in longitudinal comparison between V1 and V2 (Figure 2). The specific subcategories that significantly decreased from baseline at this dose were nasal obstruction (8.02 U decrease, 17.88%; *P*=.04), rhinorrhea (10.08 U decrease, 34.99%; *P*=.004), watery eyes (11.27 U decrease, 53.18%; *P*=.001), and itching nose (18.89 U decrease, 67.23%; *P*=.001). The symptom of itching eyes was not significantly altered by the 12-g treatment, and sneezing was increased significantly after administration of allergen (12.46 U increase, 72.02%; *P*=.002; Figure 2). We observed no significant effect on PNIF after 12 g of MSM.

**Figure 3:**
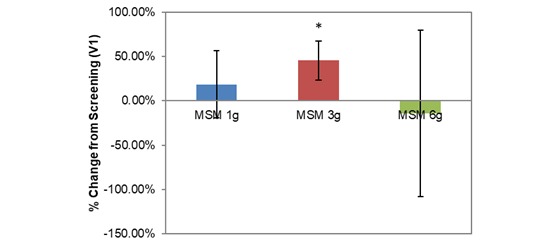
Comparison between 3 subchronic doses of methylsulfonylmethane (MSM; 1 g, 3 g, and 6 g): percentage change in peak nasal inspiratory flow from V1 (screening) to V3 (day 14). Significance was tested using linear mixed model analysis. *P ≤.05.

Longitudinal comparison between V1 and V3 across all participants produced a significantly increased PNIF with low daily MSM consumption (15.78 U increase, 17.32%; *P*=.02; Table 1). Individual low doses of MSM (1 g, 3 g, and 6 g) had variable effects on each SAR symptom when compared with baseline analysis, although each dose resulted in statistically significant decreases in at least 4 of the 8 VAS nasal symptom end points. Interestingly, when analyzed individually, only the 3-g MSM dose resulted in a significant increase in PNIF of 42.22 L/min from V1 to V3 (45.35%; *P*=.01; Figure 3).

**Table 1:** Longitudinal (V1-V3) comparison of peak nasal inspiratory flow (PNIF) and visual analog scale (VAS) average nasal symptoms across all participants (n=18).

Measure	Visit (V)			
	1 (screening)	3 (day 14)	Percentage change (V1-V3)	*P* value^a^
Number of tests	126	112	—	—
PNIF (L/min), mean (SD)	91.1 (4.315)	106.89 (4.817)	17.32%	.02
VAS score, mean (SD)	26.47 (22.950)	12.25 (12.00)	–53.72%	.001

^a^Significance was established using the linear mixed model; significant at *P*<.05.

**Figure 4:**
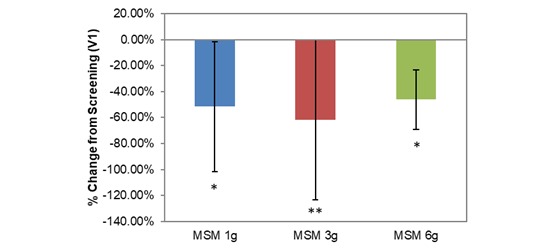
Comparison between 3 subchronic doses of methylsulfonylmethane (MSM; 1 g, 3 g, and 6 g): percentage change in visual analog scale average nasal symptoms from V1 (screening) to V3 (day 14). Significance was tested using linear mixed model analysis. *P ≤.05; **P ≤.01.

**Figure 5:**
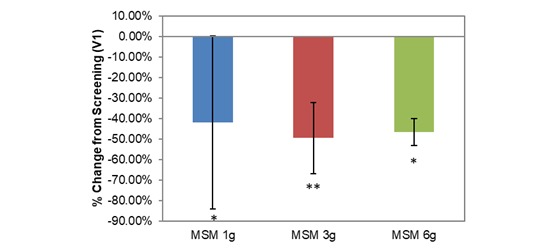
Comparison between 3 subchronic doses of methylsulfonylmethane (MSM; 1 g, 3 g, and 6 g): percentage change in visual analog scale nasal obstruction from V1 (screening) to V3 (day 14). Significance was tested using linear mixed model analysis. *P ≤.05; **P ≤.01.

**Figure 6:**
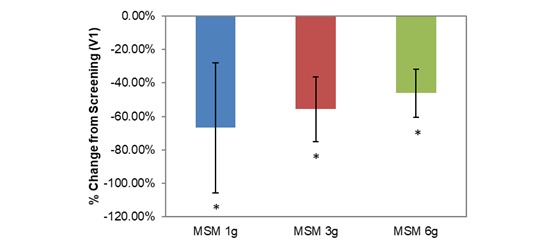
Comparison between 3 subchronic doses of methylsulfonylmethane (MSM; 1 g, 3 g, and 6 g): percentage change in visual analog scale rhinorrhea from V1 (screening) to V3 (day 14). Significance was tested using linear mixed model analysis. *P ≤.05.

VAS average nasal symptoms decreased significantly at all doses in the longitudinal comparison between visits. Across all participants, the decrease was by 14.22 U (53.72%; *P*=.001; Table 1). VAS scores decreased by 1152 U, 18.71 U, and 12.30 U for MSM 1 g (51.49%; *P*=.03), 3 g (61.65%; *P*=.009), and 6 g (46.11%; *P*=.03), respectively, from screening to V3 (Figure 4). Analysis by symptom revealed statistically significant decreases in all dose conditions for nasal obstruction for MSM 1 g (18.38 U decrease, 41.96%; *P*=.04), 3 g (25.86 U decrease, 49.59%; *P*=.009), and 6 g (17.98 U decrease, 46.62%; *P*=.02) from screening to day 14 (Figure 5). Rhinorrhea symptoms were similarly decreased at all dose levels of MSM: 1 g (16.71 U decrease, 66.86%; *P*=.03), 3 g (18.57 U decrease, 55.71%; *P*=.04), and 6 g (12.95 U decrease, 46.10%; *P*=.04) from screening to V3 (Figure 6).

**Figure 7:**
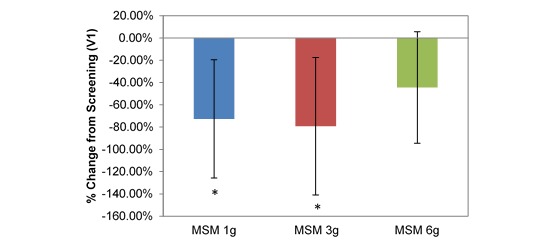
Comparison between 3 subchronic doses of methylsulfonylmethane (MSM; 1 g, 3 g, and 6 g): percentage change in visual analog scale watery eyes from V1 (screening) to V3 (day 14). Significance was tested using linear mixed model analysis. *P ≤.05.

**Figure 8:**
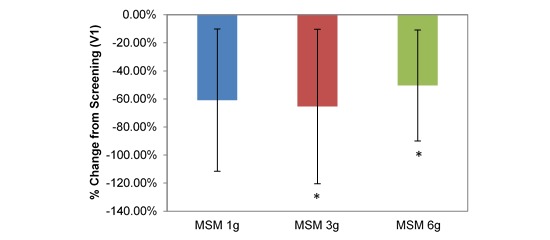
Comparison between 3 subchronic doses of methylsulfonylmethane (MSM; 1 g, 3 g, and 6 g): percentage change in visual analog scale itching eyes from V1 (screening) to V3 (day 14). Significance was tested using linear mixed model analysis. *P ≤.05.

**Figure 9:**
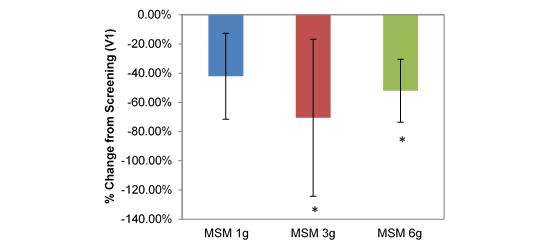
Comparison between 3 subchronic doses of methylsulfonylmethane (MSM; 1 g, 3 g, and 6 g): percentage change in visual analog scale itching nose from V1 (screening) to V3 (day 14). Significance was tested using linear mixed model analysis. *P ≤.05.

**Figure 10:**
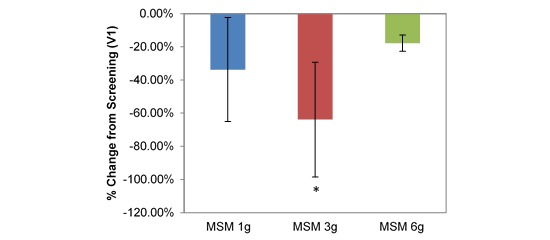
Comparison between 3 subchronic doses of methylsulfonylmethane (MSM; 1 g, 3 g, and 6 g): percentage change in visual analog scale sneezing from V1 (screening) to V3 (day 14). Significance was tested using linear mixed model analysis. *P ≤.05.

The response of the remaining SAR symptoms varied between doses. Although symptoms decreased in all dose categories, watery eyes were significantly relieved only at low doses of MSM: 1 g (13.67 U decrease, 72.66%; *P*=.02), 3 g (20.00 U decrease, 79.25%; *P*=.01), and 6 g (8.67 U decrease, 44.39%; *P*=.09) from screening to day 14 (Figure 7). Itching eyes were significantly relieved by high doses but not the lowest dose: 3 g (13.10 U decrease, 65.48%; *P*=.03) and 6 g (12.48 U decrease, 50.38%; *P*=.03) from screening to day 14 (Figure 8). Itching nose symptoms decreased only at high doses of MSM: 3 g (20.00 U decrease, 70.59%; *P*=.02) and 6 g (18.84 U decrease, 52.05%; *P*=.02) from screening to day 14 (Figure 9). Sneezing was affected only by the MSM 3-g dose, with a decrease of 14.76 U (63.92%; *P*=.04) from screening to day 14 (Figure 10).

## Discussion

### Principal Findings

In this study, we assessed the efficacy of the MSM supplement on attenuating nasal provocation by administering a standardized allergen challenge to healthy participants to induce allergic rhinitis symptoms. Although a limited human study has been performed with MSM during exposure to allergy season [13], to our knowledge, this is the first investigation into the effects of MSM consumption on attenuating a standardized allergen designed to mimic an acute allergy attack.

Normally, the nasal cavity warms, humidifies, and filters air, which is vital for the proper functioning of the upper airway tract. These physiological functions are severely diminished by passageway obstruction due to allergen exposure [17]. Immunoglobulin E (IgE) plays a major role in mediating nasal and upper respiratory allergic response following initial allergen exposure [18]. In a typical person who has developed allergies, mast cells are coated with IgE and become cross-linked on binding of an allergen. On IgE cross-linking, mast cells in the nasal mucosa degranulate and release chemical mediators known to promote well-known symptoms, including nasal itch, nasal obstruction, watery and itchy eyes, and sneezing [19,20]. The release of preformed inflammatory mediators, such as histamine, stimulates the histamine-1 receptor on sensory nerves. Stimulation of these nerves causes vascular dilation and increased plasma leakage, which results in nasal discharge and congestion [21].

This study tested the protective effects of MSM on the allergy attack following exposure to a highly concentrated nasal allergen. The allergen challenge consisted of a standardized mixture of the most common outdoor and indoor allergens that are associated with nasal allergy. We tested several doses of MSM and compared their effect with baseline symptoms, where the allergenic challenge was presented with no MSM study product present. We assessed efficacy by comparing symptoms observed after standardized allergen exposure in the presence of MSM versus symptoms observed at baseline.

An acute high dose of MSM (12 g) was administered at the clinic 30 minutes prior to the allergenic challenge. Although PNIF was not significantly altered, the acute dose of 12 g of MSM resulted in statistically significant decreases in all nasal symptoms of allergic rhinitis, except for itching eyes. This suggests a potential improvement in quality of life via improvement in symptoms experienced by the participants. This result suggests that a 12-g dose of MSM administered just before and during allergen exposure is effective in reducing patient’s stuffy nose symptoms, but it did not directly improve nasal breathing.

After the high-dose treatment visit, participants were randomly assigned to a lower daily dose (1 g, 3 g, or 6 g). They took this dose daily for 14 days, and then again 30 minutes prior to the allergenic challenge. The dosage routine was designed to mimic what might be at-home daily use of the MSM. This regimen appeared to be effective at significantly reducing most symptoms of allergic rhinitis. The MSM 3-g dose appeared most effective (consistent), significantly reducing all VAS nasal symptoms scales.

Nasal obstruction resulting from the presence of allergen or infection decreases the maximum flow of air through the nose [22]. Improvement in PNIF generally signifies improvement of nasal airway patency and corresponds to a lesser extent of nasal obstruction, which results in improved nasal breathing [23]. The results of the study suggest that, while an acute high dose (12 g) of MSM significantly improved stuffy nose symptoms of allergic rhinitis following allergen challenge, a long-term daily dose of MSM can significantly improve nasal breathing as measured by PNIF.

Our findings are consistent with those of previous MSM clinical trials. A previous multicentered, open-label trial by Barrager et al [13] assessed the safety and efficacy of orally administered MSM 2.6 g for 30 days. That study assessed environmental SAR by a seasonal allergy symptom questionnaire. Barrager and colleagues also further monitored immune and inflammatory markers, including plasma histamine, IgE, and C-reactive protein. Upper, lower, and total respiratory symptoms were significantly reduced from baseline as early as day 7, and the improvements were maintained throughout the 30-day study duration. Interestingly, there were no significant changes in levels of IgE or histamine [13], suggesting that MSM may have an alternative mode of action besides direct alteration of IgE or histamine levels. To our knowledge, our study is the first randomized, double-blind clinical trial that presented the efficacy of the MSM product for alleviation of allergic rhinitis symptoms. Here, we showed that MSM of various doses reduced the subjective and objective symptoms of allergic rhinitis in healthy participants overexposed to standardized allergens.

MSM has been shown to have anti-inflammatory properties and is reported to block the formation of inflammasomes [24]. This is in contrast to antihistamine substances that inhibit histamine production [25] and produce soporific side effects. The sulfur component of MSM may also be used by the body to produce antibodies that can combat foreign material, particularly allergens. However, there is still no reliable information to confirm the mechanism of action of MSM [26]. Animal studies have demonstrated that the anti-inflammatory effects of MSM mitigate the abnormal immune reactions that trigger inflammation [27], suggesting that a similar mechanism may be in play. Human studies have demonstrated a positive effect of antioxidant capacity MSM, which may also play a role in the mechanism of action of this ingredient under allergic rhinitis conditions [28]. Most importantly, it has been shown that MSM produces fewer side effects than prescription medications such as antihistamines [13].

The results of this randomized, double-blind study provide preliminary evidence that several dose levels of the MSM product alleviate symptoms of nasal provocation in a population of healthy participants. While the findings are promising and have produced statistically and clinically significant positive results, larger randomized, placebo-controlled trials are warranted to confirm these findings.

### Conclusion

MSM supplementation significantly alleviated participants’ symptoms in response to a standardized allergenic challenge. An acute dose of MSM 12 g was highly effective at improving the stuffy nose symptoms of allergy as measured by the VAS nasal symptom scales. A dose of 3 g of MSM daily for 14 days not only decreased all scores of the VAS nasal symptoms, but also significantly improved nasal breathing as measured by PNIF. We observed no safety concerns. More research is warranted.

## Abbreviations

IgE: immunoglobulin E
MSM: methylsulfonylmethane
PNIF: peak nasal inspiratory flow
SAR: seasonal allergic rhinitis
VAS: visual analog scale
